# Preclinical Patient-Derived Culture Models for Personalized Treatment of Pancreatic Cancer: A Dream of the Future or Useful Practice?

**DOI:** 10.3390/cancers15113027

**Published:** 2023-06-01

**Authors:** Rüdiger Braun, Hendrik Ungefroren

**Affiliations:** 1 Department of Surgery, University Medical Center Schleswig-Holstein, Campus Lübeck, 23538 Lübeck, Germany; 2 Department of Medicine I, University Medical Center Schleswig-Holstein, Campus Lübeck, 23538 Lübeck, Germany; 3 Institute of Pathology, University Medical Center Schleswig-Holstein, Campus Kiel, 24105 Kiel, Germany

Pancreatic cancer is currently the fourth most common cause of cancer-related deaths and is predicted to be the second leading cause of cancer-related mortality by the year 2030. Most of the malignant neoplasms of the pancreas are ductal adenocarcinomas (PDAC). For several reasons, the prognosis of PDAC is exceedingly poor. Surgical resection is the only curative treatment option, but only about 10–20% of patients present with resectable tumors at the time of diagnosis. Moreover, most pancreatic carcinomas are resistant to conventional (radio)chemotherapy. Current clinical standard treatment regimens comprise either mFOLFIRINOX or gemcitabine (with nab-paclitaxel). The selection of either of these regimens is currently based on the patient performance status and comorbidities, while the underlying individual tumor biology is commonly not considered. Although multiple studies aimed at establishing molecular profiles and identification of actionable molecular targets of PDACs, targeted therapies for PDAC patients have not (yet) become an integral part of clinical routine practice. In addition to treatment prediction based on molecular profiling, e.g., in the context of a molecular tumorboard, preclinical culture models that reflect the complex interactions of tumor cells with their microenvironment are promising tools to understand molecular mechanisms of PDAC and to test individual treatment responses. These patient-derived models comprise adherently growing primary cell lines, organoid cultures, organotypic slice cultures and patient-derived xenografts.

Garcia et al. recently published a comprehensive review on two-dimensional (2D) cell cultures, 3D organoid cultures, and genetically engineered mouse models (GEMMs) with a special focus on patient derived xenograft (PDX) models [1]. Adherent 2D cultures, however, have major drawbacks in that they are likely to undergo clonal drifts and expansions and to accumulate genetic changes during long-term culture, while early passage primary 2D cultures, are more heterogeneous and may contain tumor-associated fibroblasts. 3D organoid cultures in which cells are kept in suspension are derived from embryonic stem cells, induced pluripotent stem cells, somatic stem cells, or tumor cells. 3D growth in matrices that mimic a basement membrane reflects more the in vivo conditions compared to adherently growing 2D cultures. When set up as co-cultures with, e.g., stromal cells they allow heterologous cell–cell interactions to be studied. However, 3D organoid cultures still cannot fully replicate the complex cellular interactions of the tumor microenvironment (TME), particularly those with the immune cell component in PDAC. PDX models are classically generated by direct implantation of human tumor tissue into immunocompromised mice. Subcutaneous implantation requires a minimally invasive procedure and tumor growth can be monitored easily while orthotopic implantation results in tumors that resemble the primary tumor more closely and is more likely to produce metastatic lesions. Tumor morphology and differentiation as well as genetic alterations are generally maintained in the PDX models. Stromal elements albeit implanted with the tumor specimen do not expand and are replaced over time by murine stroma, which is a major limitation. Although some effort has been made to develop humanized mouse models, e.g., by transplantation of CD34+ hematopoietic stem cells, residual immune competent cells of murine origin may remain in the host, which also limits the predictive value for drug treatment. Garcia et al. discuss five studies that used PDX models of PDAC evaluating their potential utility for predicting efficacy of novel single or combination treatment regimens. However, the authors also clearly state that the value of PDX models in tailoring therapeutic regimens for individual patients is largely unknown. Data on individual therapy response prediction from PDAC-PDX and their clinical utility are very rare and were mainly derived from four studies. These studies showed unprecedented examples of patients who responded to predicted drugs. Nonetheless, the significant lag between engraftment and first passage, and a tumor take rate varying from 20 to 85% are additional limitations of PDX models in individual treatment prediction.

In light of the limitations of PDXs as a preclinical model for individual therapy response prediction in personalized medicine, 3D organoid cultures are a highly promising tool. Frappart and Hofmann comprehensively reviewed the methodology and potential use of organoid cultures to improve clinical management of PDAC [2]. Efficient and reliable cultivation of organoids has been described with a success rate of 62–100% from resected tumor specimens, fine needle aspirates, and specimens from metastases. Organoids derived from resected specimens of the primary tumor can be established in 2 to 3 weeks and a drug screen can be performed in only 4 weeks. Although the fast workflow and high success rate are major technical advantages, several methodological issues may impair reproducibility. First, the type of the cultivation matrix, e.g., collagen or solubilized basement membrane extracted from the Engelbreth–Holm–Swarm mouse sarcoma (BME2, Matrigel) and batch-to-batch variations in growth factor content or composition of the latter matrix affect growth conditions. Second, most cultivation protocols require the use of complex media including WNT-conditioned media. These also underlie batch-to-batch variations, which have been shown to impact drug response. Although some studies described the intentional incorporation of fibroblasts and immune cells into organoid cultures, these co-cultures are highly complex and their implementation in clinical routine appears to be difficult. Irrespective of these technical issues, several studies demonstrated the feasibility of drug response prediction using patient-derived organoids for PDAC patients. Particularly for gemcitabine-paclitaxel and FOLFIRINOX, a high correlation between organoid cultures and clinical response of the respective patient could be shown.

As mentioned above, most preclinical culture models lack the immune cell compartment. However, multiple suppressive immune cell types including macrophages, myeloid-derived suppressor cells (MDSCs) and regulatory T cells are found in early pancreatic lesions and persist through cancer progression. Holokai et al. described an elegantautologous pancreatic cancer organoid-immune cell co-culture model that may be able to predict the efficacy of targeted therapies [3]. Based on the observation that polymorphonuclear-MDSCs (PMN-MDSCs) are associated with poor prognosis in pancreatic cancer, the authors showed using this model that PMN-MDSCs contribute to tumor growth and the suppression of CD8+ T cell proliferation. Moreover, to address whether PMN-MDSCs impair the efficacy of checkpoint inhibition in PDAC, the same group developed an autologous tumor organoid/cytotoxic T lymphocyte (CTLs)/PMN-MDSC co-culture model. Treatment with the programmed death-1 (PD-1) inhibitor Nivolumab resulted in significantly inhibited CTL proliferation in these triple co-cultures compared to co-cultures without PMN-MDSCs. In autologous organoid/CTL co-cultures derived from PD-ligand-1 (PD-L1)-positive PDACs, organoids were sensitive to Nivolumab treatment. However, the addition of PMN-MDSCs inhibited Nivolumab-induced cancer cell death. Combined treatment for PMN-MDSC depletion and inhibition of the interaction between PD-1 and PD-L1 resulted in enhanced CTL effector function and targeting of PD-L1-expressing PDAC cells in this ex vivo organoid co-culture model. Holokai et al. thus elegantly tackled the unmet clinical need of tumor cell (organoid) and immune cell co-cultivation as an ex vivo platform to predict patients’ response to targeted therapies.

Another model system that should be mentioned in this context are organotypic slice cultures (OTSCs). We recently established this ex vivo model in our laboratory to explore its potential use for ex vivo response prediction [4]. OTSCs are a quick, cost-effective and technically feasible method for short-term cultivation of semi-intact PDAC tissue. They preserve the multicellular tissue context, i.e., tumor cells, stroma and immune cells. We detected various immune cell populations during a period of 6 days of OTSC cultivation (data unpublished). Weitz and coworkers comprehensively reviewed and discussed the use of OTSCs to study the TME of PDAC compared to other established 3D models such as organoids [5]. Distinct from other models, OTSCs retain autologous immune cells and therefore offer the unique possibility to study response of PDAC to therapeutic interventions in the preserved tissue context. Understanding tissue heterogeneity is crucial to study response to treatment, which can be assessed in the preserved tissue using immunohistochemistry and subsequent quantification by digital pathology. Furthermore, spatial molecular analyses such as proteomic profiling by MALDI-imaging allow individual therapeutic responses in the heterogenous tissue to be understood.

In summary, we conclude that to date there is no optimal model system for preclinical response prediction of individual PDAC patients to therapy. Each model system discussed here has its benefits and weaknesses and does not fully reflect the individual patient’s tumor biology in vivo. However, since they complement each other, they may be used in combination rather than singly for reliable response prediction. All of these preclinical patient-derived culture models are certainly highly promising tools in personalized treatment of PDAC; their clinical implementation, though, requires further evaluation and fine tuning.
